# Lecture on Emergencies of the Horse and Their Treatment

**Published:** 1889-07

**Authors:** R. S. Huidekoper

**Affiliations:** Brigade Surgeon, 1st Brigade. N. G. P.


### Art.—XVII.—LECTURE ON EMERGENCIES OF THE HORSE AND THEIR TREATMENT.

By Major R. S. Huidekoper,

*Brigade Surgeon, 1st Brigade. N. G. P.*

Gentlemen : A week ago I called your attention to the outlines of the anatomy of the horse. This evening I shall take up a consideration of various emergencies which you may meet with, and shall call your attention to the ordinary blemishes to which a horse is subject, that is those blemishes which offer visible symptoms, and which the unprofessional man can appreciate. I shall then take up those points in treatment which you may have to apply yourself for your own horse in the service, or which you will apply under the guidance of a professional man in the ordinary treatment of disease.

You will recall that in speaking of the anatomy of the head, I showed you the large canals, the nasal fossæ, that the greater part of the horse's head, that is the entire portion between the eyes and a line running from the eye to a point opposite the level of the grinder teeth formed a large space which is subject to inflammation, and that peculiar sac belonging to the horse and which no other animal possesses, the guttural pouches. When a horse takes an acute cold in the head, the throat or any of these three parts may be affected. Ordinarily it is a simple throat cold, but the horse may have a discharge from the sinuses in the head or from the pouches behind the throat. The discharge may come also from the tubes in the lungs or from the lungs themselves, but the immediate or emergency treatment which you can apply is the same for all.

I called your attention also to the anatomy of the poll. We have here two sets of muscles making a triangle. It is here that poll evil occurs, the first symptom of which is rapid swelling with tenderness. Such symptoms require immediate attention. The treatment, is the use of poultices or cold applications.

Coming next to the withers, we see at the top of the shoulder blade a large piece of cartilage with layers of tendons and muscles. Disease at this point is exceedingly common in horses untrained to the saddle or heavy harness, and results from the pressure of the saddle or collar. After a long ride, or on the second day of severe use, a swelling begins to appear, and later a large sore may develope in the tissues, and it then takes the name of fistula of the withers, and requires immediate treatment.

As I run my hand back over the spine, you see that this horse flinches. There is a popular error, perhaps one of the most common of popular errors, that when a horse flinches when pressed in the back, it is an evidence of kidney disease. A perfectly healthy horse always flinches when pressed in the back. A thorough-bred or well bred horse will sometimes be thrown almost to the ground by such pressure which is the normal reflex. When a horse becomes sick, suffers from kidney trouble, lung trouble or has fever, he does not flinch. This reflex sensibility is a sign of perfect health.

We next come to the shoulder. Another popular fallacy is in regard to shoulder disease or sweeny. Sweeny is a falling away of the shoulder supposed to be due to disease of the shoulder, but in reality is simply atrophy of the muscles due to want of use of the leg. In 999 out of 1000 cases, sweeny is the result of some lameness below the knee, and in the majority of cases of the foot. As a result of this the muscles are not used so much and waste, just as when a man has a felon, the muscles of the shoulder will waste. A falling away of the muscles of the shoulder can usually be considered an evidence of disease of the distal part of the leg. While shoulder lameness which belongs to the lower part of the shoulder blade and the upper part of the arm is considered to be such a common disease, you can put it to one side absolutely. One of the most difficult things in veterinary medicine is to make the diagnosis of shoulder lameness. It is generally only to be made by excluding causes of lameness in other parts of the leg. It is rare that it presents any positive signs. Where there is an injury to the shoulder causing lameness, there is inability to carry the leg forward when the attempt to do so is made.

The next point where we have a blemish is at the elbow. It is the condition popularly known as shoe boil. This is frequently seen and is due to two common causes. One is bad shoeing. A horse that has recently been to the blacksmith, in lying down curls his leg backward throwing the shoe against the elbow, causing a boil or swelling at this point. The same thing occurs when a horse unaccustomed to work is driven twenty or twenty-five miles, comes in tired and as he lies down throws the heel against the elbow causing a swelling. The condition in which you would have to treat this would be as a soft, hot, tender swelling from the size of a hen's egg to that of the fist, and which when pressed causes the horse to flinch. The immediate treatment consists simply in bathing it with cold or hot water, and if the swelling is considerable, to make half a dozen punctures into the swelling with a sharp pen-knife to allow the escape of the serum. In the course of two or three days, the treatment becomes a matter requiring a good deal of judgment, but the immediate treatment is such as I have detailed.

We come next to broken knees occurring most frequently in the saddle horse. Here the parts should be kept clean and bathed with cold water. Stitches can rarely be put into wounds at this point.

We shall next have to go back to the anatomy for a moment. You remember that below the knee we have the large canon bone and two splint bones standing vertically, the former descending to the fetlock from which the pastern passes off generally at an angle of 35°. Behind the fetlock joint are two sesamoid bones. Then we have the pastern bone, the coffin bone and the navicular bone, a sesamoid bone behind. Then we have lying behind these bones, coming off from the knee joint and fastened above the canon bone, a ligament like an elastic band which comes down dividing into two portions and fastened to the sesamoid bones on either side behind the fetlock. This has a purely mechanical action, and over it the animal has no control. When a horse is standing with the muscles at rest, the entire weight above the knee is supported by this suspensory ligament. Directly behind this band you see a tendon controlled by muscles in the fore arm. When the horse is traveling he governs the movements below the pastern joint by the tendon of these muscles. If a horse is given unusual work going beyond the power of these muscles, or is trotted down hill so that the weight is thrown forward beyond the point where the muscles can control it, these ligaments become pulled upon and may become inflamed. Again just below the knee we have a tendon serving the same purpose as the suspensory ligament. If a horse is given unusual work or trotted down hill throwing a severe strain on this part, a swelling will appear, so that a common injury is enlargement of these tendons directly below the knee.

Then, again, we get swelling of the tendons passing to the fetlock. All of these tendons are supplied with sheathes containing synovia, a lubricating fluid. When inflammation occurs these sheathes become swollen, causing what are known as wind galls. These are merely dilatations of the sheaths which lubricate these three sets of tendons passing behind the fetlock joint, and tightly bound there. Strains of any kind require prompt treatment with cold applications or excessively hot water. This is quite as efficacious as anything that you can get out of the drug store.

In connection with the bones themselves we have a trouble which is known as splint. You saw last week that the canon bone was accompanied by two rudimentary bones. The knee rests on these last above, and in the centre finds a solid support on the canon bone itself. These bones are attached by fibrous tissue. A young animal may twist the leg so that these bones become displaced, tearing the tissue, setting up inflammation and forming splints. If the horse developes properly, ossification takes place between these bones. In the majority of legs in old horses (this specimen) you see that between the canon bone and the splint bone there is perfect union. The deposit of bony matter has made a perfectly solid mass of the three bones. Up to the age of four, five or even six years, before solid union has occurred this displacement may be produced. In others (this specimen) you see that there has been a little irritation. An inch and a half of the bone is joined, but the rest remains separate.

If you find a swelling of the bone on one side of the canon bone, the horse needs rest immediately. If you keep on using him, he will throw a larger splint which may become serious. If he is given rest at once, the condition may be relieved and never give any serious trouble. If he throws a small tumor, it will make a rest of three weeks necessary. It makes a great difference on which side of the splint bones the trouble is located. If on the outer side it is only a matter of a few days rest. If on the inner side, in the gutter behind the canon bone where the tendons come down, it interferes with their free movement and may cause permanent lameness.

When we go below the fetlock we have these two bones the first and second phalanges above the hoof and one, the third, in the centre of the hoof. From a bad twist the horse may throw out a swelling at this point, the lower end of the first or on the second phalanx, which will be hot, hard and tender, making the horse limp ; it takes the name of ring bone. This may be of any size. It may be simply a growth over the bone making the parts hard and tender, or it may become as large as the fist on one side or the other of the pastern, making the animal permanently lame. Where there is simply enlargement it requires immediate rest and cool lotions, and later, more active treatment, such as blisters, or the hot iron and complete immobility of the part may be called for.

Below this, at the edges of the hoof, we have what is known as side bone or low ring bone. If you take the horse's foot in its normal condition, you will see running out on each side of the third phalanx, or coffin bone, a large piece of cartilage—the lateral cartilages they are called. When the horse puts his foot down, or when he presses on his hoof which is almost inelastic, these cartilages allow of a certain amount of elasticity. When, as a result of irritation, these cartilages become inflamed, bony deposits take place, so that when the animal steps, instead of the cartilage giving, it remains an inelastic structure, and the animal experiences pain from the pressure upon the sensitive structure of the hoof. The difference between ring bones and side bones is that one occurs over the pastern or fetlock, while the other occurs directly over the border of the coronary band or the side of it.

If we take next the hind extremity, we find that the first bony structure is the pelvis with a cavity for the articulation of the thigh bone or femur. The femur and the whole pelvis is covered by an immense mass of muscles rendering it impossible to touch bony points with the exception of the point of the haunch and the point of the ischium. No other point of bone can be felt. Taking the femur we can feel this prominence, the lesser trochanter, through an inch and a half of muscle, and below we can feel these prominences covered by muscles. The only injury that can be recognized by the non-professional man is where from a blow as from being struck by the tongue of a wagon, or a fall, the haunch bone is broken down. If broken at the end, the point of the haunch, it lays the animal up for a couple of weeks; if lower down the neck of the ilium, the lameness is permanent, although the animal may recover so far as life is concerned.

There is another lesion which is often mistaken for hip down. When you look at the horse from behind, you find that one side appears lower than the other. This is a permanent trouble equivalent to sweeny. Where a horse has a cause of lameness below, the muscles fall away. You distinguish this from hip down, by the fact that in the latter affection the bony prominences of one side are lower than on the other, while in the former the bony prominences are on the same level, but the muscles are found to be wasted.

The femur is covered by a mass of muscles four inches thick on the inner side and an inch and a half on the outer side, which are subject to a number of lesions, of which I have here some examples. Here is the pelvis and femur of a horse which by hard work has torn the attachments of the muscles. You see these large bony tumors on the under surface of the pelvis. These occurred at a point where it would be impossible to detect any lesion. The horse would be very lame in the hind legs. Take now the femur and see the number of bony prominences indicating where the muscles have been torn. Such lesions could only be detected by the professional man, and then only by examination by the rectum. The diagnosis of lameness at this point is to be made by excluding lameness due to all other causes, and by the peculiarity of the gait. The only possible treatment is absolute rest. If the cause of lameness in the hind leg is not detectable, the only thing for you to do is to tie the head up and put the animal at rest.

The next point of blemish is the stifle joint where the only lesions are luxation of the knee cap or stifle bone or inflammation of the same. The horse gives evidence of trouble here by inability to carry the leg forward and by swelling in the region of the stifle. You will remember that at the last lecture I showed you the knee cap in front of the lower part of the thigh bone secured in place by ligaments. I also showed you two ridges at the lower end of the femur, the higher being on the inner side. When this knee-cap, which you can feel move under the skin is displaced, it is thrown to the outer side causing a visible swelling. There may also be inflammation at this point. In either case, there is inability to move the leg forward on account of the intense pain produced. The part is hot, swollen and tender. Whether there is displacement or not, immediate relief is to be afforded by hot or cold applications. The pain can be relieved at once by tying the head up and pulling the leg forward by running a rope around the cannon bone between the fore legs and around the neck. When the leg is drawn forward the bones are pulled straight, the tendons are eased on and the pressure on the joint is lessened.

Immediately below this we come to an important part where injuries are very frequently met with. This is the tibia between the stifle and the hock where horses are often injured by a kick from another horse. This bone is covered by muscles on the outer side only by a dense membrane and the skin. A kick by another horse may scarcely mark the skin and cause no apparent trouble, and yet three days later the leg may snap off and the bone come through. When a horse is kicked at this point, either on the outer side or more especially on the inner side, he should at once have his head tied up and be laid off from work, for in every ten horses kicked in this way, there will be one or two, that, three or four days after the injury will have the leg snapped off. When this injury occurs, the break is a longitudinal one without displacement of the bones. Two summers ago one of the well known trotting mares was kicked in Cleveland. Was then shipped to Buffalo and jogged along for two days, and on the third day the leg broke on the race track as the result of softening. The number of cases of this accident is so great that it warrants the expense of putting every horse kicked in this way in a sling and keeping him there three weeks.

In the hock, there are three injuries which you will be apt to have as the result of accident. The first of these is swelling of the joint itself. This is commonly known as bog spavin. There is swelling on the inner side in front and on both sides behind. This is commonly caused by strain in making a jump or in pulling a heavy load, and requires immediate rest.

The other two conditions are both bone troubles. The first spavin occurs on the inner side. Taking this normal specimen you see the two rows of flat bones with the larger hock bones above. You also see here several examples of spavin. Instead of having three rows of movable bones, the inflammation has made the joint perfectly solid. Instead of the contour of the joint being that of the normal hock, with a depression here, then a slight elevation and another depression, we have here this swelling. The other curb occurs to the outer side behind, and is detected as a break in the line from the hock down, when looked at from the side.

Below this point we have exactly the same blemishes as in front. We may have inflammation of the tendons or of the bones. We have splints, but in the hind legs, splints are not so important, and are not so apt to cause lameness as in front. Coming to the fetlock, we have ring bones and side bones, and we perhaps have rather more frequently inflammation of the ligaments at the fetlock interfering with the ability of the horses to carry its rider, or to do heavy draught work. We have swellings of the superficial tissues, generally due to interfering in a horse unaccustomed to carrying a rider, or in a horse put at heavy draught work. The horse becomes tired and strikes the fetlock on one side or the other causing inflammation of these ligaments.

The foot in addition to the parts which we have already considered, consists of the wall, which is the visible part of the foot which turns around behind running in on each side to form what are known as the bars. Then we have the sole which fills in the inner side of the wall and on the outer side of the bars, we have the frog which runs to a point separated on either side from the bars. The frog is divided in the middle by a gutter, and a gutter is left on either side between the frog and the bars. In the ordinary care of the horse it is as essential as anything else that after the horse has been out, the feet should be picked up and these three clefts cleaned out thoroughly. In 99 cases out of 100, one can run his finger along this middle gutter and find that it has not been cleaned for weeks, that it is filled with decomposing matter with the smell of ammonia which will predispose to the formation of thrush. Then again nails or little stones are picked up which if removed at once cause no trouble, but if allowed to remain, will by pressure act upon the soft tissues and cause lameness. The first thing that should be done when a horse is brought in is to pick up the foot and clean out these furrows.

A word in regard to improper shoeing. Suppose you have a horse shod to-day and to-morrow he is lame. This may be either from improper shoeing or from pricking.
A horse is shod with a bar shoe and is lamer than before. A bar of the ordinary form comes entirely back, and if it presses any where it presses on the softest part of the heel where it does not afford any relief. The bar is ordinarily made convex and thus does not receive any pressure. It should be made with the point running forward and so directed as to receive the pressure of the frog.

Another trouble pretty commonly met with is pricking of the foot. The horse keeps his foot off the ground, and when forced to step on it picks it up quickly and holds it up. This requires immediate relief. You should at once have a sufficient amount of the sole cut out. The farrier usually contents himself with making a small hole where the nail is. A hole the size of a silver dollar should be made sufficiently deep to bring blood. Then a poultice or hot water dressing should be applied. The question of deeper cutting must be left to professional judgement. The emergency treatment is as I have said to have a hole the size of a silver dollar cut until you draw blood or can force it down with the thumb. If this is not done you have a dense structure, underneath which an abscess may form and cause serious trouble, while if proper treatment is adopted the horse may be used the second morning afterwards.

Having rapidly run over the more important blemishes, I shall next take up the points of treatment which you have to apply yourself or as nurses under the direction of others. The first is the treatment of colds. A horse gets an acute cold with cough and a discharge from the nose, it makes no difference whether the discharge comes from the nasal fossæ, the cavities between the eyes or from the lungs, relief is always afforded by steaming, that is allowing the horse to breath moist air which may have some medicament added to it such as pine tar, belladonna or camphor, by means of a steam bucket. The steam bucket can be used several times a day by the stable-man for fifteen minutes at a time and if done properly, with great advantage. This consists of taking a bucket half-full of bran, then mix in with the bran a handful of hay and in this way make a sponge which will hold water for some time. A little belladonna or tar may be added, but there is no necessity for anything more than the heat. If now very hot water is poured into the bucket it will give off steam for the next fifteen minutes. If you have a careful man and a quiet horse, the horse can be brought to the feed box and the attendant can hold the horse's head over the bucket. A better plan is to take a bag and cut open the lower end. One end is then placed over the animal's head and the other over the bucket and allowed to remain ten or fifteen minutes. If it is desired the steaming can be kept up longer by using hot water.

In connection with the head we have also certain other points to consider. Sometimes these sinuses have to be washed out and frequently there are troubles about the eye requiring the use of injections. Sometimes you have ordinary catarrh, a disease which has to be treated by injections. In all these procedures you must bear in mind that you have an animal that will not stand perfectly still while being treated, and you have to use more care than the physician does in treating his patients that you do not injure the animal by rudeness. In using the syringe you must always brace the end of it by the hand and wrist, so that if the animal flinches, no injury will be done. In injecting into the eye, you brace the instrument with your fingers and keep the end of the nozzle an inch from the eye.

When a horse has a bad cold, he often has swellings about the throat which have to be poulticed. When a horse has poll-evil you also have to use poultices. The average stable-man in putting on a poultice intended for the space between the jaws gets it under the throat and not where the disease is located. The proper plan is to take a cloth and fold it into a triangle, put it under the jaw and tie the ends of the triangle above the poll. This is the way that it is ordinarily put on, but in ten minutes it has worked back under the throat. To obviate this, you have to use a second bandage tied directly above the eyes, and you can fasten it to the halter to prevent slipping. If this does not hold the poultice in place take a little straw and pack it under the two bandages between the two bars of the jaw. This will throw the poultice directly into place. I venture to say that there is not more than one stable-man out of five hundred that knows how to properly apply a poultice under the jaw.

In applying a dressing to poll-evil, you can use this same triangular cloth. The ends are tied underneath and the point brought down between the ears and secured to the halter. This will keep it from slipping from side to side.

For the withers you use the same dressing and fasten to the corner on each side a piece of bandage which is run down and under the arm. If necessary the point can be fastened to a surcingle and then the dressing will remain for twenty-four hours without the slightest chance of moving.

For poulticing the foot you can use the same shaped cloth which is quite as good as any patent boot. The poultice is placed on the cloth and the foot set on the poultice. The point is brought up behind and the ends brought over each side in front cross each other, and are then tied behind, covering the point. If the animal is not restless, it will be sufficient to pin the point down, or a peice of flannel bandage may be applied outside the triangle and will hold it in place.

Suppose the horse has lost a shoe, broken its hoof and has a tender foot and you have still several miles to go. You can take two bunches of straw and braid them into two pieces. Lay them across each other on the ground with a poultice above them and place the foot on the intersection. Then bring one piece up in front and back and the others laterally. Then run a string around the pastern, turn down the ends of the braids and run the string around a second time, tie tightly and with ordinary care you can get along. The straw protects the foot and the animal can travel rapidly for several miles without disturbing the poultice and with perfect protection of the foot.

Another nice application to the withers in addition to the poultice is the ice bag. Here is an animal that happens to be in the hospital suffering from the condition to which I have referred, that is fistula of the withers. We have applied here an ice bag. The bag contains ice with a little bran to hold it. The ice bag can be applied from time to time and remain for twenty-four hours and makes a good temporary dressing. The use of this application will, if continued for a day or two, often relieve a sore pair of withers, which if allowed to continue might become a permanent trouble.

One of the most important things in the care of the horse is neglected in every stable, that is the cleaning of the sheath of the penis. When we put our hand into the sheath we usually find a mass of sebaceous matter and dirt which should not be there. It is just as essential to clean out the sheath twice a week as it is to curry the horse. This is easily done. Sometimes the horse requires the twitch. The hand is to be carried in beyond the wrist until the head of the penis is reached. At the urethral orifice you often find stones as large as large bullets or walnuts. These sometimes press upon the opening of the urethra and prevent the animal from urinating properly and may in addition lead to bladder trouble. Introducing the hand and grasping the penis, it should be pulled out and washed with soap and water. This, as I have said should be done twice a week regularly.

I wish next to say a word about colic which will occur frequently in your horses. This is not a disease in itself but simply a symptom of internal pain. A horse in the first stage of pleurisy or pneumonia will have colic. A horse that has any intestinal troubles, gets a hernia down, has indigestion or inflammation of the intestine will have colic. This is simply an internal pain. In the majority of cases of colic which comes from the abdominal troubles, whether inflammatory or simply the result of indigestion, the symptoms are more marked than where the colic comes from lung trouble or commencing heart disease. There are certain things that should be done in either case. The administration of internal remedies depends entirely upon the cause. You will have two animals with apparently the same symptoms and yet one will have an inflammatory colic requiring bleeding, while the other will have a colic from indigestion requiring the use of internal remedies without bleeding. The colic mixture that is supposed to treat every colic, while applicable in one case is not in another. One thing that should be done in any case is to clean the rectum. The arm should be soaped and carried up to the elbow and what fæces there are removed. The fingers should then be brought together and the arm coaxed in without force until it enters to the shoulder, and what matter is found removed. Care must be taken that the intestine is not torn. This will usually allow the horse to pass wind. An injection should then be given either with the ordinary syringe or with a piece of rubber tube with a funnel attached. This is elevated a couple of feet and fluid poured in. In this way a larger quantity can be introduced than with the syringe. Another method of treatment is two men and a whip. One man at the head walks the horse for a time. If the trouble still continues, start out again and if necessary use the whip. Muscular action is one of the best means of securing a movement of the bowels and one of the most prompt methods of treating colic.

There are two other troubles to which I desire to call attention. One is founder. After a day's march, you find the horse stiff and unable to move. On the outside of the bone the horse's foot is covered with a series of laminæ about one-half inch in width and almost imperceptible of thickness. Some 500 of them surround the entire foot. On the wall of the foot you see corresponding leaves one-half inch in width and with depressions. If these leaves with their secondary leaves, which cover a surface of twelve square inches were spread out on a single plane they would cover ten square feet. When a horse stands, he stands by the hold which these leaves take on each other. You can cut out the sole of the foot entirely taking off all support from the bottom and put the foot in a vice and you will be unable to force the foot through the wall. The horse does not travel on the sole, the whole weight is borne by the laminæ. Sometimes from over-heating or over-feeding, or from want of use for a few days with immediate use afterwards, or from feeding barley to a horse unaccustomed to it, the horse gets an inflammation of these structures and a little blood or serum is thrown out. This causes most intense pain and is known as founder. If the fore feet are affected the horse is found with the hind feet propped under him to take the weight of the body. If the hind feet are affected, the front feet take the weight. If all are affected, the hind feet will probably be found under, and the front feet forward. If this is allowed to go on the horse may lose the foot, or deformity with throwing of the toe upwards may be caused. The wall should be at once thinned down and the animal walked into a stream of water. In private stables the feet may be put in hot water. Founder, consists in this, congestion and effusion separating the two layers of laminæ.

The popular idea of horses being foundered by drinking when overheated is absolutely without the least foundation. It may give the animal pneumonia or colic, but it will not cause founder. In the early part of this century, when England landed the troops in Portugal to move into Spain, she landed two thousand English cavalry which had been on ship board seven days. They were moved ten miles that night and given a heavy ration of barley, The next day they were again moved ten miles after another ration of barley. By evening twelve hundred of the two thousand horses were foundered. They had here a rest of seven days with a march immediately afterwards and a heavy ration of barley. Barley must be cautiously fed to horses not accustomed to it, but heavy feeding of grain of any sort in horses not accustomed to it may cause founder.

Another trouble is staggers. This occurs in hot weather more than at any other time. There are two forms, that known as blind staggers and mad staggers or epilepsy. In the one case the horse becomes restive, may attempt to rear or run and then becomes absolutely stupid. It is impossible to back the animal. He will start to eat hay and hold it in his mouth without continuing to chew it. These symptoms may disappear to reappear in a few weeks.

Another form,—stomach staggers,—is due to the use of fresh clover. This will give violent symptoms of colic with excessive rotation of the head. Almost the only treatment is bleeding which may be done either just behind the teeth, which is hardly sufficient, or from the jugular vein which is found on the side of the neck. This, however, requires a certain amount of skill. There is no prompt internal treatment except laudunum.

In dealing with horses we have not the advantage of intelligence as in the human being, and it is necessary to employ a certain amount of contention. One of the means employed is the twitch. The twitch is made of a piece of leather rough on one side the same as is used for belts, with a loop of about this size. If you use rope, it should be about as thick as the finger. The twitch may be put on the nose or the ear. It holds the animal through pain. As the animal stands quiet there is very little pain, but if he attempts to throw his head up, it causes pain and he soon learns to keep quiet. If you want to move a horse on which you have the twitch do not attempt to lead him by the twitch, but by the halter. Sometimes you have to put on two twitches. After the twitch is removed you rub the animal's nose to get rid of the crease. Remember never to put a twitch on a horse's tongue as is so frequently done. You will frequently find the fraenum torn, or the tongue cut half through as the result of this. I warned you last week that in opening the mouth, you should not pull the tougue out.

Another means of contention is the use of hobbles. The operation consists in applying the proper hobbles, drawing the four legs together and throwing the animal on his side so that the shoulder drops first and the croup last. If the croup falls first injury may be inflicted. Even with the greatest care some horses will fracture the croup or some bone of the legs or in the greater number of cases some bone in the back. Out of one thousand cases, about three will fracture the back. Careful investigation shows that this occurs in old animals where there is disease of the bones. A sound animal does not break his back. While the animal is down the head should be held off the ground, and if the animal is violent, up. If the head is allowed on the ground, he gets a point of support and in his struggles may break his back. If you have a severe cut in the leg where you have to control hemorrhage one or other leg can be drawn up or one leg can be tied across the other. The hobbles are not always available and there is one method with a rope which may be employed. The rope should be thirty feet in length ; six feet from the end you make a loop. Run the loop around the horse's neck and make a simple tie knot below to keep the other knot from slipping ; then throw the rope under his leg and up around the arm pit. This makes a slip noose around the two front legs. Then carry the rope to the pastern point of the back foot. The rope is then drawn up on the other side. The animal is then thrown. If further contention is required the rope can be looped around the other leg and it can be drawn up in the same way and tied to the shoulder. In removing this hobble the first end of the rope is loosened and the loops taken off the hind feet and those of the front feet will loosen themselves.

Every once in a while you will have a horse injure himself where the case is perfectly hopeless. The best method that you have for destroying the horse is probably the firearm. If a butcher is convenient, there is nothing better than the poll-ax. One unaccustomed to hitting animals in the head will fail and inflict unnecessary pain. The pistol, carbine, revolver or musket will be most convenient for you. The barrel should be directed immediately under the ear. If the aim is in front there is a tendency to shoot too high and the ball strikes between the eyes in which case it has to go through the sinuses before it reaches the brain. A ball directed two inches below the ear will destroy the horse with as little pain as any method. There is another which is known as pithing, in which a sharp-pointed instrument is driven into the poll. The horse drops perfectly motionless, so quickly that the operator has to take care that he is not caught. This requires a certain amount of experience. The fire arm is absolutely certain to destroy the animal instantly without pain.

